# Fracture Resistance of Endodontically Treated Teeth Restored With Short Fiber Reinforced Composite and a Low Viscosity Bulk Fill Composite in Class II Mesial-Occlusal-Distal Access Cavities: An Ex-vivo Study

**DOI:** 10.7759/cureus.42798

**Published:** 2023-08-01

**Authors:** Harish Selvaraj, Jogikalmat Krithikadatta

**Affiliations:** 1 Conservative Dentistry and Endodontics, Saveetha Institute of Medical and Technical Sciences, Chennai, IND

**Keywords:** endodontically treated teeth, sdr flow+, everx posterior, low viscosity bulk fill composite, fiber reinforced composite

## Abstract

Background

Teeth undergoing endodontic therapy are prone to structural weakening and increased risk of fractures. The absence of marginal ridges and pericervical dentin further compromises the fracture resistance. The choice of the post-endodontic coronal seal is crucial for the effectiveness of endodontic therapy.

Aim and Objectives

This study compared the fracture resistance and fracture modes of endodontically treated teeth (ETT) with mesial-occlusal-distal (MOD) cavities restored with two different posterior composite resins: e-glass fiber reinforced composite (FRC) (everX Posterior) and flowable bulk fill composite (SDR Flow+).

Materials and Methods

Sixty human maxillary first bicuspids were divided into four groups: Group PC- positive control (intact teeth), Group NC- negative control (unrestored endodontically treated teeth), Group EXP- samples restored with everX Posterior and nano-hybrid composite, and Group SDR- samples restored with SDR Flow+ and nano-hybrid composite. The NC, EXP, and Smart Dentin Replacement (SDR) samples underwent endodontic procedures and MOD cavity preparation. The samples from EXP and SDR groups were restored with composite resins as post-endodontic coronal seals. The fracture resistance was evaluated using a Universal Testing Machine (UTM), and fracture modes were examined under a dental operating microscope (DOM) at 6x magnification. Statistical tests were performed using One-way ANOVA and Tukeys' post hoc tests.

Results

The mean fracture resistance of the experimental groups was as follows: PC- 880.1 ± 209.3 N; NC- 238.1 ± 15.4 N; EXP- 766.1 ± 50.2 N; SDR- 540.8 ± 49.4 N. The highest fracture resistance values were observed in the PC group, whereas the NC group showed the least. The EXP group exhibited significantly higher fracture resistance than the SDR group. Adhesive failure was observed in most samples in the EXP group, whereas samples in the SDR group showed more cohesive failures. Favorable fractures were more prevalent in samples restored with EverX posterior.

Conclusion

The study findings suggest that everX Posterior can enhance the fracture resistance of structurally compromised ETT. Samples restored with everX Posterior showed a favorable mode of fracture, which can be restored. Applying FRCs can contribute to the longevity and success of endodontic treatment by reinforcing the weakened tooth structure and preventing fractures.

## Introduction

The loss of structural integrity driven by access preparation can be attributed to an upsurge in endodontically treated teeth (ETT) fractures, leading to increased cuspal deflection during function [1]. A mesial-occlusal-distal (MOD) preparation has reduced relative cuspal rigidity by 63% while eliminating one marginal ridge has reduced tooth rigidity by 46% [2]. Recently, the role of peri-cervical dentin (PCD) is also implicated in influencing the resistance to fracture of ETT teeth. This region is located about 4 mm, superior to the crest of the alveolus. Notably, a significant portion of the PCD cannot be regenerated or replaced once lost. Excessive axial reduction in conventional full coverage post-endodontic restorations leads to a significant loss of peri-cervical dentin [3]. Loss of proprioception in ETT can also impair the protective reflexes in preventing transmission of occlusal load [4]. Considering these factors, post-endodontic restoration's role is pivotal for endodontic therapy's outcome. 

The role of bonded restorations with fiber-reinforced composites has been extensively researched in the literature. Evolution in bonding agents has improved the quality of adhesives and biomimetic bonded restorations, which reduces microleakage and improves marginal seal [5,6]. Among these, the short fiber reinforced composite (SFRC) everX Posterior (EXP) has evolved to replicate the strain-enthralling characteristics of natural teeth. Reinforcing fibers enhances the internal strength of structurally-compromised teeth and prevents fractures [7]. To overcome the difficulties of adaption, low-viscosity bulk fill composites (LVBF) called Smart Dentin Replacement flow+ (SDR) with enhanced mechanical characteristics have been developed as an alternative to high-viscosity resin composites. SDR technology includes a patented urethane dimethacrylate structure introduced to reduce polymerization shrinkage [8].

There is currently no existing literature comparing the fracture resistance of everX Posterior and SDR Flow+ as core materials in MOD access preparations of ETT. Hence, this study aimed to compare the fracture resistance and modes of fracture of endodontically treated teeth (ETT) with MOD cavities restored with EXP and SDR.

## Materials and methods

The study obtained prior clearance from the institutional human ethical committee (IHEC/SDC/ENDO/-2005/21/281). Sixty human maxillary first bicuspids (sample size achieved by G*Power 1-β = 90%, α = 0.05) [9] with closed apex, extracted due to orthodontic reasons, were acquired from the department of oral and maxillofacial surgery, Saveetha Dental College & Hospital, Chennai, Tamil Nadu, India. Upon examination under magnification and transillumination, these teeth were free from caries and showed no evidence of prior cracks, fractures, or restorations. Calculus and soft tissue deposits were carefully cleaned using an ultrasonic scaler (Acteon Satelec P5; Wyboston, United Kingdom). A thorough water rinse followed this, and the teeth were kept in de-ionized water and used for the experiment within a month.

Base preparation of samples

To simulate the alveolar bone and periodontal ligament (PDL), a layer of molten wax, approximately 0.1 to 0.2 mm thick, was applied to the roots of all samples, from 0.5 mm below the cementoenamel junction (CEJ) to the apex. Specially designed molds with standard dimensions of 6.5*6.5 cm were created for holding the tooth samples. PMMA resin (DPI; Mumbai, India) was manipulated and poured into the mold, and samples were vertically placed onto the resin, positioned 0.5 mm beneath the CEJ. After the resin had been set, the wax was eliminated from the mold cavity using a spoon excavator. The mold cavity was packed with low-viscosity silicone elastomer (Zhermack, Italy), and the samples were then reseated. The extruded flash was trimmed using a no. 15 scalpel blade (Amkay; Thane, India).

**Figure 1:**
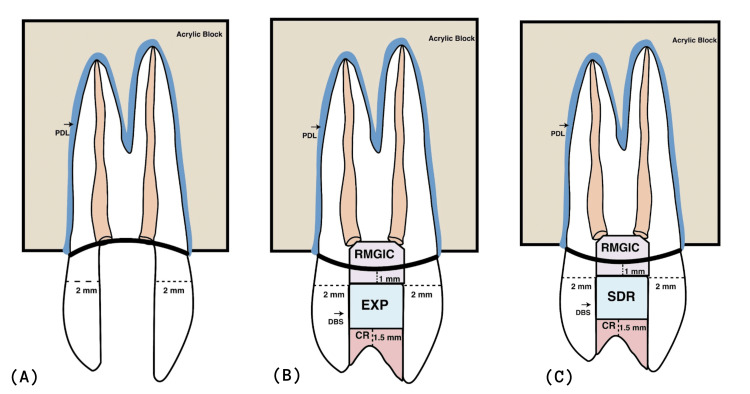
Illustration of sample preparation (A) Unrestored tooth (B) After conditioning, coronally restored with EXP followed by an occlusal layer of hybrid composite, (C) After conditioning, restored with SDR followed by an occlusal layer of hybrid composite RMGIC- Resin Modified Glass Ionomer Cement; PDL- Periodontal Ligament; EXP- EverX posterior. Image Credits: Dr. Harish Selvaraj.

The samples were randomly divided into four groups (n = 15) for the study. The positive control group (Group PC) consisted of intact teeth without endodontic therapy; groups NC, EXP, and SDR were subjected to endodontic procedures and MOD (mesial-occlusal-distal) cavity preparations. The negative control group (Group NC) (Figure 1A) represented ETT which was left unrestored. Group EXP used everX Posterior (GC EUROPE) for dentin replacement and nano-hybrid composite resin for enamel replacement, and Group SDR utilized SDR Flow+ (Dentsply DeTrey; Konstanz, Germany) for dentin replacement and nano-hybrid composite resin for enamel replacement.

Endodontic treatment of samples (Groups NC, EXP, SDR)

In 45 teeth, access for endodontic therapy was prepared conventionally using a high-speed airotor hand-piece under water coolant (NSK Inc. Japan), with the aid of a small-sized round bur BR-31 (Mani Inc., Japan). The pulp chamber was deroofed and refined using ultrasonic tips (Start X, Dentsply, Germany). Canals were worked to the apex with a #10 K file. The working length of the canals was calculated by deducting 1 mm of the length achieved. The crown-down approach was used for root canal preparation with Dentsply pro taper gold rotary files (Dentsply Maillefer, Switzerland). The torque and speed settings were adjusted per the manufacturer's recommendations. Root canals were intermittently cleansed using a 30-gauge side-vented needle and 2 ml of 5.25% sodium hypochlorite (NaOCl) solution (Parcan, India). For each new file, 2 ml of 5.25% NaOCl and 2 ml of 17% ethylene diamine tetra-acetic acid (EDTA) solution (Endo Solution, Cerkamed, India) were used for irrigation. Final irrigation was carried out using 5 ml of 5.25% sodium hypochlorite, followed by rinsing with distilled water and drying the canal with paper points (Meta Absorbent Paper Points, Meta Biomed, Korea). Up to #25 tip size and 0.06 taper was used as master apical file size for biomechanical preparation of the root canals. After drying the canals, they were filled with GP-Points 25/0.06 (ProTaper Gold Conform fit, Dentsply Maillefer, Switzerland) coated with sealer (AH Plus, Dentsply Maillefer, Switzerland). Excess obturation material on the canal orifice was removed using hot hand pluggers, and the chamber was cleaned with cotton dipped in pure ethanol (Vetro Power; Mumbai, India). A radiographic evaluation was done to examine the status of the obturation.

The Mesial-Occlusal Distal (MOD) cavities were prepared using carbide burs FG 245 (SS White; New Jersey) with a high-speed air rotor hand-piece under water coolant. The specific measurements for the cavity dimensions were as follows: (1) Buccal wall - 2 mm, (2) Buccal CEJ (cementoenamel junction) - 1.5 mm, (3) Palatal wall - 2 mm, and (4) Palatal CEJ - 1.5 mm. A periodontal probe was used to estimate the cavity dimensions during the preparation. The palatal and buccal walls were prepared parallel to each other.

1 mm of Resin-Modified Glass Ionomer (RM-GIC) material (Fuji II LC, GC EUROPE) was applied to the floor of the pulp chamber. The access cavities were restored using the two designated restorative materials, EXP and SDR, per their assigned groups. AutoMatrix (Dentsply Sirona, USA) created the proximal contours during the restorative procedure.

Post-endodontic restoration

Composition of Materials Tested in Study

EverX posterior (everX Posterior GC EUROPE) - It is composed of a resin matrix, fibers of e-glass that are randomly aligned, and inorganic particle fillers. TEGDMA, PMMA, and Bis-GMA make up the resin matrix.

SDR flow+ (SDR; Dentsply-Italy) - Modified UDMA, TEGDMA, butylated hydroxytoluene, and ultraviolet stabilizing agent are all components of the resin matrix. The filler component comprises silanated glass, surface-treated fumed silicas, silanated strontium aluminum fluorosilicate, and ytterbium fluoride.

Group EXP

The enamel margins were conditioned by applying an etchant gel (Prime Dental Products; India) for 15 seconds and rinsed with water. The cavity walls and floor were blow-dried gently. Using micro applicator tips, Single Bond Universal (3M ESPE) was meticulously rubbed on the walls of the cavity and light-cured for 20 seconds using Bluephase N (Ivoclar Vivadent, India). A nano-hybrid composite Z350XT (3M ESPE) was used to build the proximal walls with a thickness of 1 mm. The everX posterior (GC EUROPE) material was incrementally applied, allowing a 1 mm area to restore the occlusal portion, which was completed using Z350XT composite (3M ESPE), with each layer being light cured for 20 seconds. The occlusal surface was polished using medium to ultra-fine aluminum oxide polishing discs (Shofu Super Snap Rainbow Kit) (Figure 1B).

Group SDR

The same conditioning protocol as that of Group EXP was followed. A nano-hybrid composite Z350XT (3M ESPE) was used to build proximal walls with a thickness of 1 mm. SDR Flow+ (SDR; Dentsply-Italy) was applied in 4 mm increments, allowing 1 mm area for the restoration of the occlusal portion, which was completed using Z350XT composite (3M ESPE), with each layer being light cured for 20 seconds. The restoration used the medium to ultra-fine aluminum oxide polishing discs (Shofu Super Snap Rainbow kit) (Figure 1C).

Fracture resistance testing

Each sample underwent 10,000 cycles of thermocycling in a fluid chamber at 37 degrees Celsius [10]. To simulate chewing forces over approximately one year, cyclic compressive loads ranging from 15 to 30 N were applied on the palatal incline of the buccal cusp. This was achieved using a 2.5 mm steel ball at a cross-head speed of 0.5 mm/min. A total of 250,000 cycles were completed using a Universal Testing Machine (UTM) (Instron 9400, USA), simulating nearly one year of chewing [11].

Following the cyclic loading, a steel sphere with a width of 6 mm was used to apply compressive force on the buccal and palatal cusps, as well as the surface of the restoration of each test specimen. The force was continuously applied until the specimen was fractured, and the fracture resistance was measured in newtons (N). Subsequently, the samples were examined under a dental operating microscope (DOM) (Carl Zeiss Opmi Pico, Zeiss AG, Germany) at a magnification of 6x to identify the mode of fracture and determine whether it was favorable or unfavorable.

Statistical analysis of the fracture resistance

The mean values among groups were compared using one-way ANOVA, and Tukey's HSD post hoc tests for multiple pairwise comparisons with (p-value <0.05) were considered statistically significant.

## Results

Positive controls (Group PC) exhibited the highest fracture resistance values, 880.1 ± 209.3 N, and the least fracture resistance values were observed in the negative control group (Group NC), 238.1 ± 15.4 N. The mean fracture resistance of the samples was 766.1 ± 50.2 N for EXP (Group EXP) and 540.8 ± 49.4 N for SDR (Group SDR) (Table 1). A statistically significant difference was observed between EXP and SDR groups which favored the EXP group (p<0.05) (Table 1).

**Table 1:** Mean and Standard Deviation of the peak load of fracture among groups in Newtons (N) EXP: everX posterior+nanohybrid composite; SDR: Smart Dentin Replacement flow plus+nanohybrid composite; PC: positive control; NC: negative control, * indicates One-factor ANOVA.

Groups	Mean ± Std. deviation	p-value
PC	880.1 ± 209.3	<0.05*
NC	238.1 ± 15.4	
EXP	766.1 ± 50.2	
SDR	540.8 ± 49.4	

Eleven samples from EXP (group EXP) and five samples from SDR (group SDR) showed adhesive failure (Table 2). All the samples restored with EXP experienced favorable fracture (fracture line above the level of CEJ), whereas ten samples from the SDR group experienced an unfavorable mode of fracture (fracture line below the level of CEJ).

**Table 2:** Mode of Failure EXP: everX posterior+nanohybrid composite; SDR: Smart Dentin Replacement flow plus+nanohybrid composite

Groups	Number of Cohesive Failures	Number of Adhesive Failures	Number of Mixed Failures
EXP	4	11	0
SDR	10	5	0

## Discussion

The structural integrity of ETT is weakened during access cavity preparation. Pulp removal during routine endodontic therapy eliminates a helpful positive feedback mechanism, increasing the risk of tooth fractures [2]. Endodontic therapy results in a 5% reduction in tooth stiffness, while a 69% reduction is attributed to structurally compromised tooth structure due to caries, trauma, or previous restorative procedures. The loss of the marginal ridge significantly decreases a tooth's fracture resistance [1]. The investigation focused on MOD cavity design in ETT teeth, which exhibited substantial cuspal deflections [12].

Furthermore, premolars, with their anatomical characteristics including shape, crown-root ratio, and crown volume, were chosen as they are more prone to cusp fractures, representing the worst-case scenario [12]. The experimental setup considered most of the parameters relevant to the clinical scenario. To adequately evaluate the fracture resistance of the restorative material and understand its impact on stress distribution, it was crucial to incorporate the periodontal ligament and bone in the laboratory testing protocols. This inclusion ensured a comprehensive assessment of the material's ability to withstand fractures and accurately represented the physiological conditions of stress distribution [13]. As a previous study [9] recommended, a layer of elastomeric impression material was used to simulate the periodontal ligament, effectively reducing the occlusal pressures exerted on the specimens. The samples underwent thermocycling and dynamic loading to replicate the aging and masticatory process observed in clinical scenarios [11].

The SDR technology features a proprietary urethane dimethacrylate structure known for its ability to reduce polymerization shrinkage, a significant problem associated with bulk-fill composites. This polymerization modulator helps decrease the stress buildup that naturally occurs during light polymerization [14]. Another approach to mitigate polymerization shrinkage in composites involves employing a slower polymerization rate of the resin. This contributes to enhanced flowability, reduced stress accumulation, and improved interfacial integrity, particularly in flowable composites [15]. Using larger filler particles with an average size of 4.2 μm in SDR flow+ compositions reduces the number of contacts between the filler and matrix. This reduction in interfaces results in less interference and light scattering, leading to improved translucency, light penetration, and curing characteristics [16]. SDR flow+ demonstrated increased bond strength and lower polymerization shrinkage stress values, reducing cuspal deflection [17]. Therefore, SDR flow+ was chosen for comparison with EXP in this study. However, the majority of the samples in the SDR group underwent an unfavorable mode of fracture and exhibited lower fracture resistance values compared to the EXP group. The increased creep values associated with flowable composites could be attributed to the effect of the lower level of cross-linking in the organic matrix [18]. The lower fracture resistance values observed in the SDR-restored teeth groups, as well as the occurrence of unfavorable fracture when compared to the EXP group, could be explained by the lower elastic modulus of SDR compared to EXP [19]. Similar results were observed that EXP exhibited superior fracture resistance in endodontically treated teeth when used as a core material, compared to other particulate filler composite resins [9,11].

Direct restorations were considered a conservative option for restoring ETT. The use of reinforced composites, which possess properties similar to natural dentin, along with advancements in adhesive systems, has led to more favorable clinical outcomes with direct composite restorations [20,21]. Dentin exhibits two toughening mechanisms, intrinsic and extrinsic, contributing to fracture resistance. Extrinsic mechanisms, such as crack bridging, micro-cracking, and crack blunting, reduce the magnitude of stress and increase the energy required for fracture [11]. EverX posterior, a short fiber-reinforced composite (SFRC), mimics the fibrous structure of dentin [22,23] and consequently exhibits increased fracture resistance and flexural strength [24]. Fibers exceeding the critical length of 0.5 mm-1.6 mm significantly modify stress dynamics through mechanisms like crack deflection and bridging, effectively preventing crack propagation.

Additionally, these composites efficiently transfer stress from the matrix to the fibers, preventing crack formation [24]. In the resin matrix or at the particle interfaces, interparticle crack propagation is the primary cause of fracture. EXP's linear filler particles guide the crack away from the direction of the highest stress [23,24]. This crack deflection process effectively decreases the stress intensity factor, allowing particles near the crack to form a bridge. Elastic bridging is facilitated by the ability of the reinforced fibers to bend, while frictional bridging involves friction between the fibers and their surroundings [11].

Apart from their enhanced mechanical characteristics, these composites also exhibited anisotropic polymerization contraction behavior, contributing to the reduction of polymerization shrinkage. The interpenetrating matrix was believed to absorb any remaining contraction stress during polymerization. EXP was claimed to have a polymerization shrinkage of 0.17%, which reduced the shrinkage stresses associated with bulk-fill composites [25,26]. The enamel replacement of the occlusal 1.5 mm with nano-hybrid composites prevented crack propagation through the interface, resulting in a polishable surface and improvements in marginal integrity, surface texture, and anatomical form. These bi-layered restorations acted as a mono-block, dispersing stresses throughout the tooth's long axis [24]. Using a bilayered restoration that mimicked the structural inhomogeneity of natural dentin significantly enhanced crack resistance [23]. This finding held particular clinical significance for EXP as it demonstrated the ability to improve the biomechanical characteristics of teeth with reduced resistance to fracture by emulating the toughening process found in natural dentin [22-24,27]. This combined biomimetic approach and the superior properties of SFRC resulted in increased fracture resistance in structurally compromised MOD cavities in the EXP group. Most samples restored with EXP exhibited an adhesive mode of failure, indicating that the interface between the tooth and restoration was the weakest point. In contrast, most samples in the SDR group exhibited cohesive failure due to the high bonding strength between the adhesive and SDR, surpassing their strength. 

Limitations include a relatively small sample size and the assessment of fracture resistance using only two composite materials. Future research should address these limitations by comparing fracture resistance among composite resins in various cavity configurations and including molar teeth subjected to high masticatory load.

## Conclusions

Within the limitations of this study, the e-glass short fiber reinforced composite EXP demonstrated increased fracture resistance compared to SDR and could be used to restore endodontically treated teeth in high-stress bearing zones. The random fiber orientation of e-glass fillers in EXP effectively prevented polymerization shrinkage, crack propagation, and evenly distributed stress. The samples restored with EXP also exhibited a favorable mode of fracture that could be restored. However, future studies should investigate the fracture resistance of other teeth, such as molars, when restored with FRCs as post-endodontic coronal restorations under different cavity configurations.
